# Introducing Point-of-Interest as an alternative to Area-of-Interest for fixation duration analysis

**DOI:** 10.1371/journal.pone.0250170

**Published:** 2021-05-10

**Authors:** Nak Won Rim, Kyoung Whan Choe, Coltan Scrivner, Marc G. Berman

**Affiliations:** 1 Masters in Computational Social Science, The University of Chicago, Chicago, Illinois, United States of America; 2 Department of Psychology, The University of Chicago, Chicago, Illinois, United States of America; 3 Mansueto Institute for Urban Innovation, The University of Chicago, Chicago, Illinois, United States of America; 4 Department of Comparative Human Development, The University of Chicago, Chicago, Illinois, United States of America; 5 Institute for Mind and Biology, The University of Chicago, Chicago, Illinois, United States of America; 6 Grossman Institute for Neuroscience, Quantitative Biology and Human Behavior, The University of Chicago, Chicago, Illinois, United States of America; University of Tübingen, GERMANY

## Abstract

Many eye-tracking data analyses rely on the Area-of-Interest (AOI) methodology, which utilizes AOIs to analyze metrics such as fixations. However, AOI-based methods have some inherent limitations including variability and subjectivity in shape, size, and location of AOIs. In this article, we propose an alternative approach to the traditional AOI dwell time analysis: Weighted Sum Durations (WSD). This approach decreases the subjectivity of AOI definitions by using Points-of-Interest (POI) while maintaining interpretability. In WSD, the durations of fixations toward each POI is weighted by the distance from the POI and summed together to generate a metric comparable to AOI dwell time. To validate WSD, we reanalyzed data from a previously published eye-tracking study (n = 90). The re-analysis replicated the original findings that people gaze less towards faces and more toward points of contact when viewing violent social interactions.

## Introduction

Since the pioneering works of Buswell [1] and Yarbus [2], eye-tracking has increasingly become an important method in answering a variety of questions in diverse disciplines such as psychology, neuroscience, marketing, and computer science [3–6]. The eye-movement data from eye-tracking provide a rich source of complex data that can be analyzed through a variety of methods. Currently, the majority of these methods are based on Area-of-Interest (AOI; also called Region-of-Interest or ROI) analyses. AOIs are defined as areas in the stimulus space relevant to the research question and could be used to analyze a variety of eye-movement metrics such as fixations, saccades, or scan paths [7–9].

The popularity of AOI-based methods comes from its interpretability and its capability to investigate phenomena in stimulus space. For example, AOI dwell time [7] is calculated by summing the duration of fixations that landed within the AOI. The resulting metric can be interpreted as the amount of time the participant gazed at the area in which researchers are interested. Statistical tests such as analysis of variance can then be applied to examine if there are statistical differences between different conditions or between AOIs.

Various methods for defining AOIs have been suggested [10]. One approach is to draw shapes (e.g., ellipse, rectangle, circle) around the objects of interest. Shapes used for AOI definitions in this approach vary between and within studies. For example, Scrivner et al. [11] defined AOIs for faces by drawing ellipses around the face, Lazarov et al. [12] used rectangles for defining face AOIs, and Võ et al. [13] used rectangular AOIs for mouths while using ellipses for faces, eyes, and noses. Another approach for defining AOIs is to draw custom shapes that follow the shape of the object in interest. For example, Tatler et al. [14] drew custom boundaries to define AOIs for various body parts and objects. A third approach is to segment the stimulus into grids and treat each grid as a separate AOI that could be associated with an object of interest (e.g., [15]).

This variability in AOI definitions has led to valid criticism of AOI-based methods. Although some researchers have suggested guidelines in defining AOIs [7, 16–18], there is no gold standard for defining AOIs. In addition, although methods that automatically generate AOIs have been put forward [10, 19–23], the dominant approach in eye-tracking studies is to manually define AOIs. Therefore, researchers often make subjective decisions in defining AOIs, causing locations, shapes, and sizes of AOIs to vary even between studies that utilize similar stimuli [10, 24]. Combined with the fact that researchers rarely make their AOI definitions public [25], this variability and subjectivity could potentially make inter-study comparison difficult and decrease the reproducibility of studies.

**Fig 1:**
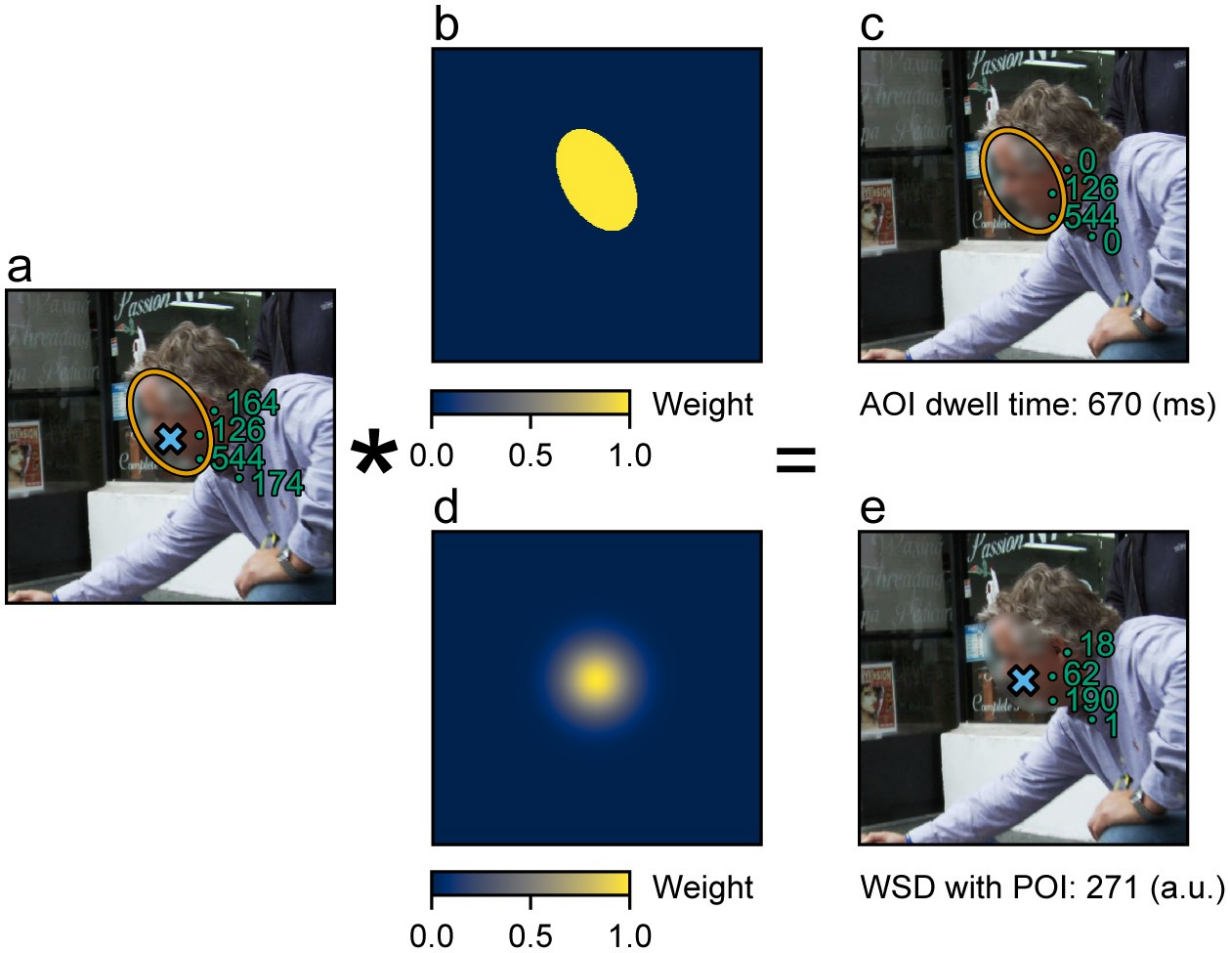
Example of AOI dwell time and WSD calculation. a) An example of a participant’s fixations (Bluish-green dots where bluish-green numbers denote the fixation duration for each fixation), AOI (orange ellipse) definition, and POI (sky blue X) definition are shown on a subset of an exemplar image. Although we present a partially-blurred image here to protect privacy, the participants saw real, unblurred images in the experiment. b) The uniform kernel weights used for AOI dwell time calculations. c) An example of AOI dwell time calculation. d) The Gaussian kernel weights used for the WSD calculation. e) Example of WSD calculation. Note that the original fixation durations have been reweighted based on their proximity to the POI.

Another inherent problem with the AOI approach is that it can exacerbate the effect of video-based eye-tracking errors [26–28]. AOI-based methods classify fixations into dichotomous classes—the fixation resides within an AOI or it does not. This is problematic because there will be fixations that reside very close to the boundary of an AOI (see Fig 1c for an example), and the inclusion and exclusion of these fixations become almost arbitrary considering the measurement errors. In other words, small measurement errors that could make a fixation cross an AOI boundary will have a large effect on the overall dwell time since the inclusion and exclusion of fixations is decided by a hard decision boundary. Moreover, this dichotomous classification treats all fixations equally as long as the gaze resided within the AOI. In other words, this method does not take into account that a fixation located closer to the center of the AOI likely has a higher probability of being related to the object of interest than fixations located very close to the AOI boundary.

In an attempt to address methodological issues with the AOI method, various alternative methods have been suggested. One of the most common alternative methods is fixation map analysis and its variations [29, 30], where the location of each fixation and a metric related to each fixation (e.g., fixation duration) are mapped onto a three-dimensional space. Fixation maps provide an intuitive visualization of fixation dispersions and have been used for illustrative purposes in various studies. For example, with this method one can create heatmaps that locate fixations and color-code them by their duration, using hotter colors to signify longer fixation durations.

However, an important drawback of these methods is that it is difficult to apply statistical tests to access significant differences within the stimulus space (i.e., it is hard to quantify whether participants are differentially looking at different parts of the image). Because of this, many studies use methods such as fixation maps for visualizations but still rely on AOI-based methods to run their statistical tests and to draw their conclusions (e.g., [14]). It is worth noting that a toolbox (iMap) that does not use AOIs has been proposed to address this issue [24, 31]. However, this toolbox needs a normalized space between all stimuli for it to work properly (analogous to MRI images being normalized into standard atlas spaces like the Montreal Neurological Institute template so that all individual participant’s brains can be compared to each other by being in the same space), which limits the types of eye-tracking studies with which it can be used. For example, this method could be applied to analyze eye-tracking data where participants looked at a variety of human portrait images since most human portrait images will have common components, such as eyes that would appear in similar positions in the stimulus space. However, it would be difficult to apply this method to stimuli that would be challenging to place in a normalized space such as having participants look at a set of abstract art, where there may not be common features that appear in similar positions across art pieces.

In this study, we propose a new method, which we call Weighted Sum Durations (WSD) analysis, that allows for fixation duration analyses while decreasing the variability of AOI definitions but retaining the interpretability of AOI dwell time analysis. This method utilizes Points-of-Interests (POIs), defined as single-pixel points in the stimulus space, as an alternative to AOIs. This substitution reduces the variability of AOI definitions by a large margin since POIs are defined only in terms of locations while AOIs are defined in terms of shape, size, and locations. Furthermore, we demonstrate that the POIs can be defined in a data-driven fashion so that the location of POIs will not rely fully on subjective decisions. Although the dimensionality of AOI definition is reduced, the semantic meanings (e.g. faces) are still maintained by the POIs upon definition, allowing them to retain much of the interpretability of AOIs.

To calculate a dwell time-like metric, our method weights the duration of fixations by the distance between the fixation and the POI and sums them to produce a single metric for the POI (i.e., the WSD). Specifically, an isotropic Gaussian kernel centered at each POI is used to weight the fixation durations. Importantly, the Gaussian kernel shares the same shape and size across all POIs, reducing the subjectivity and variability issues that plague the AOI method. Additionally, this approach naturally circumvents the problem of AOIs as dichotomous classification since there are no hard boundaries in this method and all fixations are weighted differently by their distance to the POIs. As an illustration of WSD analysis, we applied the POI-based method to a study that used AOI dwell time analysis [11]. We show that the results of this study can be replicated using WSD.

## Materials and methods

### Overview

This study used a previously collected eye-tracking dataset with 72 images and 90 participants [11]. This dataset is publicly available and can be downloaded from the Center for Open Science (https://osf.io/sfyj2/). Scrivner et al. [11] used AOI dwell time analysis for statistical testing. In this study, we analyzed whether the results of this study can be replicated using WSD analysis instead of the AOI dwell time analysis. Although we used data from a study previously published in a peer-reviewed journal, this work does not constitute dual publication since we are applying a novel analysis method to replicate the results from the previous study. The results using the AOI dwell time method are only presented in this paper to allow for easy comparison between the conventional method and our newly proposed method. Although we provide a brief explanation of the dataset below, please see the original study for a more detailed explanation of the experimental design and the data collection process.

### Participants

Ninety participants participated in the study (86 completed demographics survey; median age = 20; 56 self-identified as females and 30 self-identified as males). All participants had normal or corrected-to-normal vision (with contacts) and spoke fluent English. Informed consent was provided and signed from all participants in the study. The experiment was approved by the Social Sciences Institutional Review Board at the University of Chicago and all procedures were executed in accordance with the relevant regulations and guidelines.

### Materials

#### Stimuli

Seventy two colored images depicting interactions between two adult males were shown to the participants in random order. All images were 1600 x 900 pixels and were collected from various media sources. One-third of the images (24 images) displayed violent interactions between two adult males, one-third of the images displayed friendly interactions between two adult males, and one-third displayed ambiguous (not clearly violent nor friendly) interactions between two adult males.

#### Apparatus

Participants sat 95 cm away from a 24-inch LCD monitor. The resolution of the monitor was 1920 x 1080 pixels, and the images were displayed at the center of the screen in their native resolution. Sixty pixels corresponded to a visual angle (VA) of 1°. MATLAB with the Psychophysics Toolbox extension [32–34] was used to present the stimuli. Eye movements were recorded from both eyes via an SR Research (Ottawa, Ontario, Canada) Eyelink 1000 eye tracker with a sampling rate of 500 Hz using head free-to-move remote mode. The eye tracker was calibrated using a nine-point calibration routine and validated for all participants individually before the experiment.

### Procedure

Each participant went through a practice block and four main blocks. Practice blocks used 6 images that were not included in the study. The 72 images for main blocks were randomly split into 4 blocks of 18 images for each participant. In each trial, an image was presented for 6 seconds, and participants were asked to look at the image naturally. After the image presentation, participants rated the degree of violence in the shown interaction using a 7-point Likert scale with ‘1’ indicating *not violent* and ‘7’ indicating *extremely violent*.

At the start of each trial, participants had to click a small dot with a diameter of 0.3° (18 pixels) that appeared at the center of the screen. The central dot served as an implicit required fixation location [35] where the participants had to fixate their gaze to aim and click the mouse cursor [36]. Since gazing at the central dot in the pre-stimulus period carried over to the first fixations, this allowed Scrivner et al. [11] to check the quality of the eye movement data at the trial level and to drift-correct the eye movement data based on the first fixations of each trial.

### Eye-tracking data processing

#### Preprocessing

The data were preprocessed using the Eyelink Data Viewer (SR Research) to acquire discrete fixation locations and the duration of each fixation. All first fixations were excluded from analysis since these fixations were carried over from clicking the central dot prior to the image being displayed.

#### Offset-correction and drift-correction

The monitor used in this study had a resolution of 1920 x 1080 pixels, while the images presented had a size of 1600 x 900 pixels. Since images were presented in their native resolution, the coordinate of each fixation from the preprocessed data was corrected to account for this offset.

In addition to the offset-correction, Scrivner et al. [11] also accounted for the video-based eye-trackers measurement error by drift-correcting the fixation locations based on the location of the first fixations in each trial. Specifically, the coordinate of the first fixation of each trial was considered to be the coordinate of the central dot, and the difference between the two was corrected. The direction of drift-correction was mostly within the 90°/270° axis and 45°/225° axis (S1b Fig in S1 File). The mean magnitude of drift-corrections across all trials was 1.32° (79.39 pixels; SD = 1.39°; S1c Fig in S1 File).

#### Discarded trials

All trials that had total fixation time (excluding the first fixation) less than 3,000 ms (half of the display time) were discarded to rule out trials with potential measurement errors or trials where participants were inattentive to the image. Furthermore, trials that had drift-corrections greater than 3 standard deviations from the mean were discarded from the analysis. In total, 292 trials across all participants (4.5%) were discarded and 52 participants had no discarded trials. On average, a participant had 3.24 discarded trials (SD = 5.77, median = 0, max = 23).

### AOI dwell time and WSD analysis

#### Calculation of AOI dwell time and WSD

AOI dwell time was calculated by summing the duration of all fixations located within the AOI. This is equivalent to applying uniform kernel weights to fixation durations based on their corresponding fixation locations (Fig 1b) and summing them. WSD was also calculated by applying kernel weights to fixation durations and summing the results, but isotropic Gaussian kernels centered at the POI coordinates were used instead (Fig 1d). In other words, bigger weights were applied to fixation durations where fixation locations were closer to the AOI. The isotropic Gaussian kernel for each POI was constructed using a bivariate Gaussian probability density function with mean as POI location and isotropic covariance matrix in the form of $\left[\begin{array}{cc} \sigma^{2} & 0\\ 0 & \sigma^{2} \end{array}\right]$ (refer to later sections for how POIs and the *σ* were chosen for the WSD analysis). The kernel was divided by its maximum value so that the weights were normalized to [0, 1], and the weights were rounded to three decimal places.

#### AOI definitions

**Fig 2:**
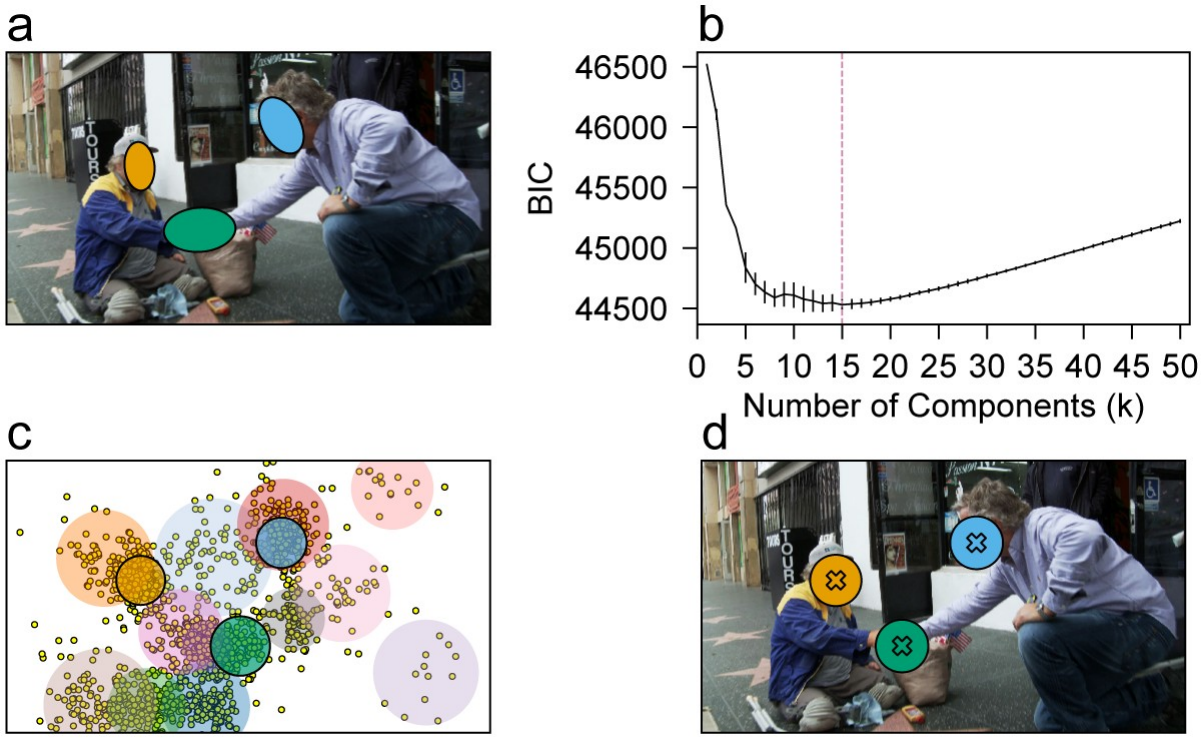
Example of AOI/POI definitions and selected mixture components. a) AOI definitions from the original study (ellipses) are illustrated in an exemplar image. b) The average BIC for GMMs fitted with different numbers of components (*k*). The error bar denotes the standard deviation of BIC across 50 different fitted GMMs. The reddish-purple dotted line denotes the number of component with the least average BIC. c) An example of visualization of Gaussian components of the fitted GMM with the selected number of components that had the lowest BIC. The yellow dots represent the offset- and drift-corrected locations of all fixations across all participants to the exemplar image. The components are visualized as semi-transparent colored circles with means at the means of the components and radii of 2*σ*. The components selected to match the AOI have black boundary lines. d) An example of selected mixture components that correspond to the AOIs defined in the original study. The means of selected mixture components (Xs) were used as the POI.

Scrivner et al. [11] defined three types of AOIs for their analysis—faces, points of contact, and objects. They defined the AOIs by drawing ellipses surrounding the objects of interest (see Fig 2a for an example). Since dwell time on the object AOIs were not significantly related to any results in the original study, we only conducted analyses using the face AOIs and point of contact AOIs without any modification from the original study. The face AOIs were defined for all 72 images while point of contact AOIs were defined for the 37 images that contained contact points.

#### POI definitions and determining the *σ* of the WSD Gaussian kernel

Since each POI serves as the center for the weighting kernel, the optimal position of a POI will be the center of a fixation cluster. Building on this, we defined the POIs for our analysis using the bivariate Gaussian Mixture Model (GMM) [37, 38], which is a model used for a variety of tasks such as clustering and density estimation. Although the *k*-means clustering algorithm is more widely used in clustering analysis of fixation data [39–41], we chose to use the GMM method since it allows us to estimate the densities of fixation as well, not just cluster membership. GMMs assume that the data are generated from a mixture of random and normally distributed components; each with a unique mean and variance. The data are bivariate in our case (i.e., each fixation location has an x-coordinate and a y-coordinate), therefore each Gaussian component will have a 2 x 1 column vector of means and 2 x 2 covariance matrix. The mean vector and the covariance matrix of each individual Gaussian component can be estimated using algorithms such as the Expectation-Maximization (EM) algorithm [42], which we utilized for fitting GMMs in this study. This setting is quite similar to WSD which weights the fixations based on a Gaussian kernel centered at POIs, making GMM potentially a good tool to guide POI definitions and to estimate the covariance of the Gaussian kernel for WSD analysis.

Because the Gaussian kernel for WSD analysis is designed to be isotropic, we used the spherical GMM, a special form of GMM that restricts the covariance matrix to be isotropic [43]. In other words, all Gaussian components in spherical GMM have a covariance matrix in the form of $\left[\begin{array}{cc} \sigma^{2} & 0\\ 0 & \sigma^{2} \end{array}\right]$ (i.e., no covariance between the x- and y-coordinates). For each image, we aggregated all offset and drift-corrected fixation locations for that image across all participants. As we were lacking strong theoretical justification for the optimal number of components (*k*) to initialize (i.e., we knew that there will be faces of two interacting adults in the image, which fixations will likely be clustered around, but we could not justify that those faces are the only part of an image that will draw fixations), we used a grid search for *k* ∈ {1, …, 50} using the Bayesian information criterion (BIC) [44] as the evaluation metric (Fig 2b) for each image. Specifically, 50 spherical GMMs were fitted using the EM algorithm for each number of components, and the number of components that showed the least average BIC was used. The mean number of componets used for GMM fitting was 12.63 (SD = 2.52, min = 6, max = 19; S2 Fig in S1 File)

After determining the number of mixture components, we visualized the Gaussian components of the fitted GMM with the chosen number of components that had the lowest BIC (Fig 2c). We then identified components that semantically matched each AOI from Scrivner et al. [11] and used the mean of the identified component to define the POI for that AOI (Fig 2d).

When we were unable to find Gaussian components that matched a previoulsy defined AOI from Scrivner et al. [11], implying that the fixations were not clustered near that AOI, we defined the POI as the mean of the AOI from the original study. No face POI was defined in this manner while 10 point of contact POIs were defined in this manner. Finally, the *σ*’s of all Gaussian components corresponding to AOIs were averaged to use as the *σ* value for the WSD analysis. The averaged *σ* value was 45.01 pixels (SD = 13.04 pixels), equivalent to 0.75° in visual angle (S3 Fig in S1 File). Building on this, we used 0.75° (45 pixels) as the *σ* for the WSD analysis. To illustrate the weighting, a fixation point that was located 1° away from the POI received approximately 0.41 weight and a fixation point that was 2° away from the POI received approximately 0.03 weight.

#### Calculation of AOI/POI saliency

The physical saliency of each pixel was calculated using the Graph-Based Visual Saliency algorithm [45]. Then, the same kernel weights used in the AOI dwell time calculation (Fig 1b) and the WSD calculation (Fig 1d) were applied to the physical saliency of each pixel and summed to calculate the physical saliency of AOIs and POIs. The physical saliency of AOIs and POIs was normalized by the total saliency of each image and was included in all statistical analyses to control for the physical saliency of AOIs and POIs.

### Linear Mixed-Effects Models

Trial-level Linear Mixed-Effects Models (LMM) were fitted to both AOI dwell time data and WSD data. LMM can isolate the effect of interest while controlling for the differences between participants and stimuli [46]. To control for the difference between participants and stimuli, random intercepts were included for stimulus and participant (i.e., they were random effects) for all models. When the outcome variable was AOI dwell time, the LMM included the saliency of AOIs and the size of AOIs as fixed effects to control for the two. When the outcome variable was WSD, the LMM included the saliency of the POIs as a fixed effect. We did not include the size of POIs in the model since POIs and Gaussian kernels used in WSD calculations all had the same size. All model statistics (*b* estimates, confidence intervals, *t*-values, *p*-values, marginal *R*^2^, and conditional *R*^2^) are reported in S1 Table in S1 File.

### Robustness to noise

To investigate how AOI dwell time and WSD differ when the level of noise increases, we repeated the analysis after systematically adding noise to the drift-corrected fixation locations. Specifically, we generated Gaussian noise separately for horizontal and vertical coordinates and added that noise to each trial’s fixation location (similar to [47]). This method was chosen since eye-tracking devices generally produce white noise even when artificial eyes were used for recording [48, 49]. Then, the AOI dwell times and the WSDs were calculated from this altered dataset with the added noise using the same AOI and POI definitions used in the main analysis. Finally, LMMs investigating the relationship between violence rating and fixation durations on faces, which was the weakest relationship in the original study, were fitted to the data. This process was repeated 1,000 times for 4 different levels of noise (four different standard deviations of Gaussian noise: 0.25°, 0.5°, 0.75°, 1°). We then investigated how many times the tested relationship reached significance with three different significance levels (*α* = 0.01, 0.05, 0.1). The Gaussian noise was generated using NumPy [50]’s random module, and models that failed to converge were excluded from the analysis.

### Code availability and software acknowledgment

All codes used for the data analysis, including the Python functions that can be generalized to use for other eye-movement datasets, can be downloaded from https://osf.io/wgma5/. SciPy [51], pandas [52], and NumPy [50] packages in Python3 were used for general data processing and analysis, including the calculation of WSD. The GMM fitting and BIC calculation was performed using the GaussianMixture Class from scikit-learn [53] package in Python3. The LMM was fitted using lmerTest [54] package built on top of lme4 [55] package in R [56]. The tidyverse [57] package was also used for general data manipulation in R. For visualization, matplotlib [58] package in Python3 and ggplot2 [57] package in R were used. The colorblind-friendly color template from Wong [59] and Brewer [60] was used for color selection. Finally, MATLAB (The MathWorks, Natick, MA) was used for extracting the AOIs from the original dataset and calculating the physical saliency of images using the GBVS algorithm [45]. The script from the GBVS algorithm was downloaded from http://www.vision.caltech.edu/~harel/share/gbvs.php.

### Supplementary analysis

We also repeated the analysis setting full width at half maximum of *σ* to 2° (i.e. *σ* = 0.85°; S2 Table in S1 File). This value corresponds with the size of foveal vision, which is about 2° in diameter [61–64] and was a value used for fixation map analysis in previous studies [65, 66]. In addition, we also conducted an additional analysis setting all POI definitions to the center of an AOI ellipse, rather than using GMMs, with *σ* = 0.75° (S3 Table in S1 File) and *σ* = 0.85° (S4 Table in S1 File). The results were not substantially different from those reported in the main article.

## Results

### Correlation between AOI dwell time and WSD

**Fig 3:**
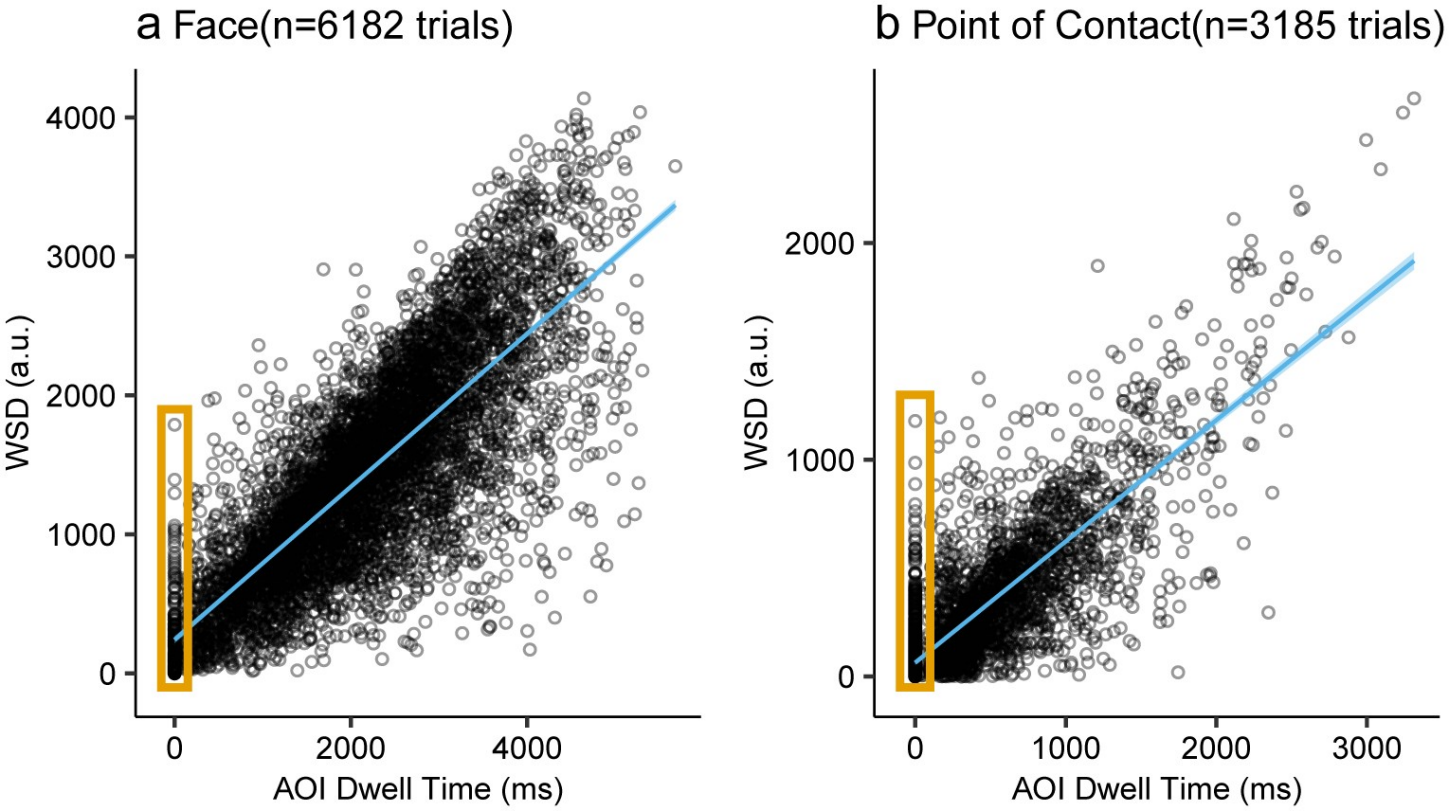
Correlation between AOI dwell time and WSD. a) Correlation between dwell time on face AOIs and face WSDs. b) Correlation between dwell time on point of contact AOIs and point of contact WSDs. The sky blue line denotes the fitted regression line. The shaded region denotes the 95% confidence interval (due to the large number of data points, it is not very visible). Each point denotes a trial. The orange rectangle highlights the trials with zero AOI dwell time. The number of trials for b) is smaller than a) because there are fewer stimuli with point of contact AOIs/POIs while all stimuli had faces AOIs/POIs.

Dwell time on AOIs and WSD of POIs were highly positively correlated on both face AOIs/POIs (r(6180) = 0.80, *p* <.001; Fig 3a) and point of contact AOIs/POIs (r(3183) = 0.81, *p* <.001; Fig 3b). Interestingly, 18.68% of the trials using images with point of contact AOI (595 trials out of 3185 trials) had zero point of contact AOI dwell time but had non-zero point of contact WSD. In contrast, only 3.15% of trials (195 trials out of 6182 trials) had zero face AOI dwell time but had non-zero face WSD. This suggests that point of contact AOIs could have neglected a large number of fixation points that were sufficiently close to POI to get weight in the WSD analysis compared to Face AOIs.

### Replication of the original study

We tested if the WSD analysis is robust enough to replicate the three main findings of Scrivner et al. [11]. Analogous to the original study, trial-level LMMs were used to investigate the relationship between the outcome variable and predictor variable in all analyses. Note that we excluded some outlier trials based on the magnitude of drift-correction, which was not accounted for in the original study, so the reported statistics could deviate slightly from the results reported in the original study.

#### Interaction type and fixation durations on faces

**Fig 4:**
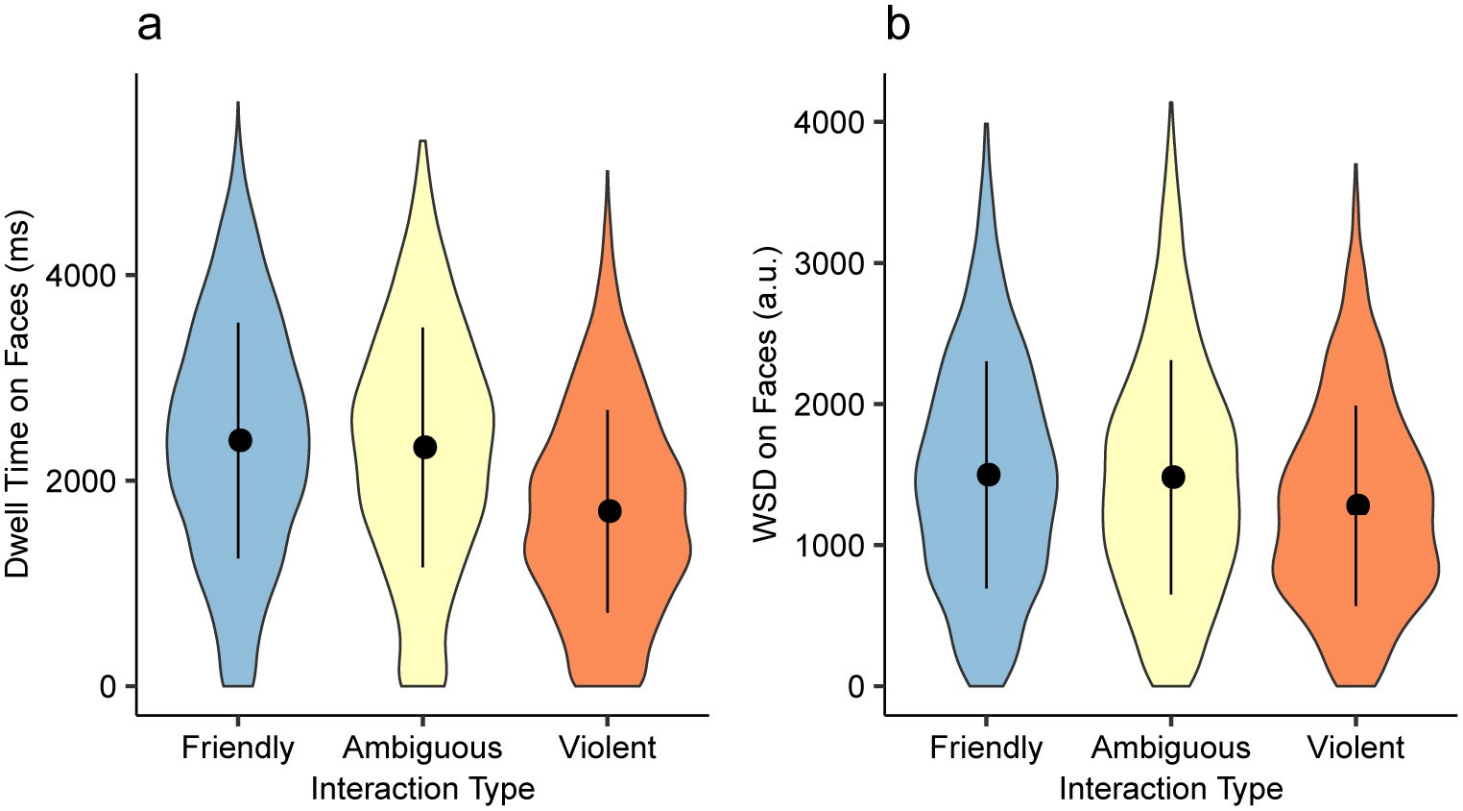
Fixation durations on faces by interaction type. a) AOI dwell time on Face AOIs by predefined interaction types. b) WSD on Face POIs by predefined interaction types. Error bars represent ±1 SD. Although the values look similar, WSD and AOI dwell time uses different metrics and cannot be compared directly.

The first major finding of Scrivner et al. [11] was that participants showed less dwell time on face AOIs when looking at images showing violent interactions compared to when they were looking at images showing friendly interactions or ambiguous interactions. As a baseline, we first fitted LMMs using dwell time on face AOIs as the outcome variable and the depicted interaction type in images as the predictor variable. Analogous to the results from the previous study, we found that participants fixated significantly less inside face AOIs when the interaction shown in the image was violent than when it was friendly (*b* = -360.49, 95% CI [-615.62, -105.38], *t* = −2.807, *p* = .006) or ambiguous (*b* = −327.58, 95% CI [-580.33, -74.85], *t* = −2.575, *p* = .012; Fig 4a). Next, we used WSD for face POIs as the outcome variable and depicted interaction type in the images as the predictor variable for the LMM. We found that participants showed significantly less WSD for face POIs when looking at images showing violent interaction compared to looking at images showing friendly interaction (*b* = −167.37, 95% CI [-327.41, -7.33], *t* = −2.077, *p* = .041) or looking at images showing ambiguous interaction (*b* = −170.62, 95% CI [-328.08, -13.17], *t* = −2.153, *p* = .035; Fig 4b).

#### Violence rating and fixation durations on faces

**Fig 5:**
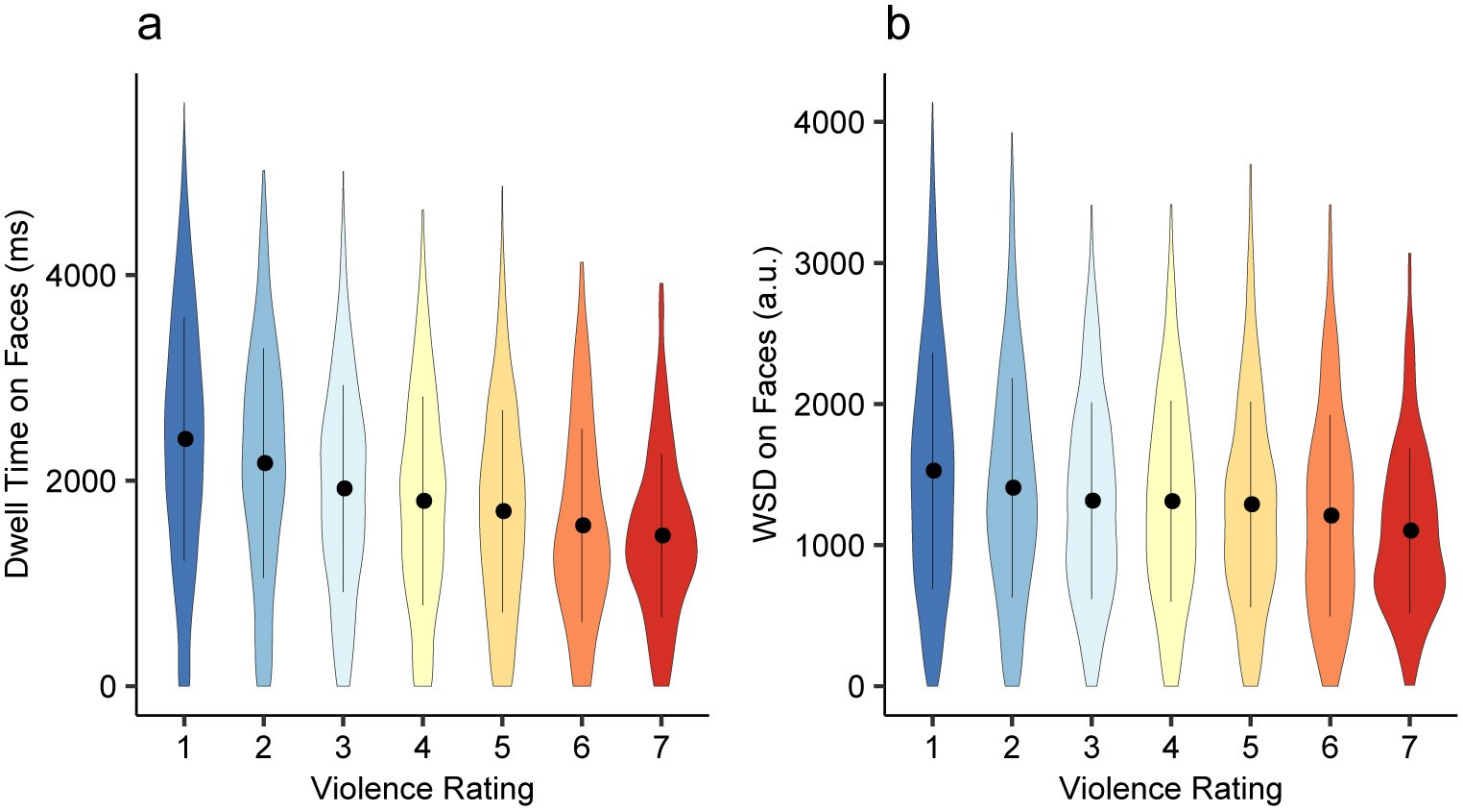
Fixation durations on faces by violence rating. a) AOI dwell time on Face AOIs by violence rating determined by individual participants. b) WSD on Face POIs by violence rating determined by individual participants. Error bars represent ±1 SD. Note that for the LMM analysis violence ratings were z-scored within participants, but the original ratings were shown here for illustrative purposes. Although the values look similar, WSD and AOI dwell time uses different metrics and cannot be compared directly.

The second main finding of Scrivner et al. [11] was that participants showed less dwell time on face AOIs for images they rated as more violent. We first attempted to replicate this finding by fitting an LMM using violence rating given by participants as the predictor variable and AOI dwell time on faces as the outcome variable. To account for the individual difference in the standard for violence judgment, we z-scored the violence rating within participants. In line with the result using interaction type as the predictor variable, participants spent less time fixating inside the AOIs when they rated the depicted interaction more violent (*b* = −54.66, 95% CI [-102.96, -5.95], *t* = −2.253, *p* = .024; Fig 5a). Furthermore, z-scored violence rating was a significant predictor variable in LMM using WSD on face POIs as the outcome variable (*b* = −37.80, 95% CI [-72.75, -2.35], *t* = −2.157, *p* = .031; Fig 5b).

#### Interaction type and fixation duration on points of contact

**Fig 6:**
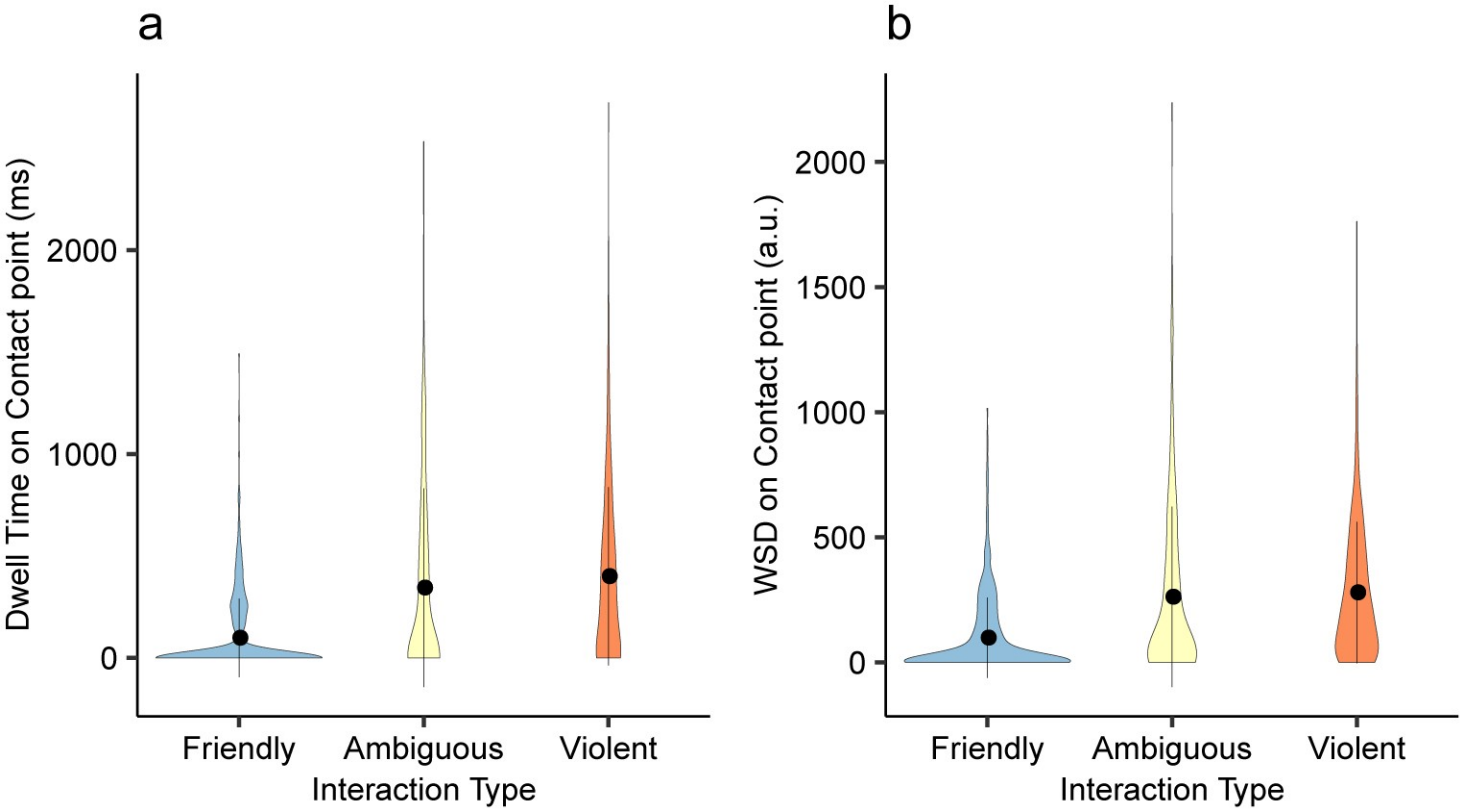
AOI dwell time and WSD on point of contact by interaction type. a) AOI dwell time on points of contact by predefined interaction type. b) WSD on points of contact by predefined interaction type. Error bars represent ±1 SD. Although the values look similar, WSD and AOI dwell time uses different metrics and cannot be compared directly.

The third finding of Scrivner et al. [11] was about participants’ dwell time on point of contact AOIs when looking at images with all three AOIs in the image (face, point of contact, object held by a person). When viewing these images, participants showed increased dwell time on point of contact AOIs when looking at violent interactions compared to when looking at images with friendly interactions. We tested this effect by fitting LMM using interaction type as the predictor variable and dwell time on point of contact AOI as the outcome variable. In line with the results from the original study, we found that participants fixated significantly longer on the point of contact AOIs when viewing violent images that contained all three AOIs compared to when viewing friendly images that contained all three AOIs (*b* = 285.68, 95% CI [110.85, 460.54], *t* = 3.477, *p* = .005; Fig 6a). Furthermore, we tested if this effect replicated if we used the WSD on point of contact POIs as the outcome variable. In the 12 images that contained all three AOIs defined, participants’ WSD on point of contact POI was significantly higher when viewing violent images than when viewing friendly images (*b* = 177.28, 95% CI [41.68, 312.90], *t* = 2.782, *p* = .017; Fig 6b).

#### The Effect of noise on subsequent linear models

We calculated AOI dwell times and WSDs on the altered data with added Gaussian noise and fitted LMMs using AOI dwell time or WSD on faces as the outcome variables, and z-scored violence rating as the predictor variable. Out of 8,000 LMMs fitted to the data from 4,000 generated datasets (1,000 datasets for 4 different Gaussian noise distributions where sigma was manipulated for 4 different levels), 187 LMMs (2.34%) failed to converge and were excluded for analysis (101 used AOI dwell time as the outcome variable, 86 used WSD as the outcome variable). The mean *p*-value for the LMMs fitted using AOI dwell time was higher than that of LMMs fitted using WSD across all noise levels (S4a Fig in S1 File). Additionally, the proportions of LMMs that showed statistically significant relationships were higher for LMMs fitted using WSD compared to LMMs fitted using AOI dwell time across all noise levels and all significance levels (S4b Fig in S1 File). Similar results were shown when we use added noise to each fixation or each participant instead of each trial. These results suggest that WSD was less affected by added noise than AOI dwell time.

## Discussion

We developed and validated a point-of-interest-based method for fixation duration analysis, Weighted Sum Durations (WSD), by replicating three main results from previous research [11] which used AOI dwell time analysis [7]. Given that WSD is robust enough to replicate the results from AOI dwell time analysis, we suggest that WSD could be a valuable alternative approach since it has some advantages over both AOI-based approaches and approaches that do not use AOIs. WSD analyses decrease the subjectivity and variability of AOIs [10, 16, 24, 25] by utilizing POIs instead of AOIs. Moreover, the POI approach still follows the basic framework of AOI-based approaches to provide a metric that can be directly substituted for AOI dwell time for statistical testing in the stimulus space.

Furthermore, the WSD approach does not use a hard boundary that classifies fixations dichotomously as being in or out of the AOI but instead uses a soft boundary that down-weights fixations that are far from POIs. This is advantageous over the AOI dwell time approach since this can potentially mitigate adverse effects of video-based eye-tracking errors [26–28] for fixations located in positions where it is difficult to judge whether the fixation is related to the object of interest or not. In other words, the effect of small measurement errors that could make a fixation cross an AOI boundary will have less of an effect in the WSD approach since there is no hard boundary. Additionally, by up-weighting fixations that are closer to POIs and down-weighting fixations that are further from POIs, researchers can take into account the probability of a fixation being related to the object of interest more directly.

POIs could also be useful when objects in the stimulus space make up a small portion of the image or do not have natural boundaries. For example, Scrivner et al. [11] defined AOIs for points of contact. As the word “point” implies, this object of interest is inherently centered to a single point, and it becomes very difficult to decide on where to draw the boundary of the AOI. As a result, there was a large number of trials (942 trials or 29.58%) with zero AOI dwell time for the point of contact. However, in a large proportion of these trials (595 trials or 63.16%) had non-zero WSDs for these points of contact. Our results suggest that using the POI approach can be a great alternative when we can be sure of the central point of an object, but uncertain of where the discrete boundary of the object lies.

Another advantage of WSDs over AOI dwell times is that POIs are easier to store and share than AOIs. There is no standard programming language or data structure in defining and storing AOIs. Combined with a relatively large amount of information needed to define AOIs, this means that researchers often need to learn new programs and data structures and convert these idiosyncratic data structures into a format they are familiar with to access the AOI information defined by other researchers. On the contrary, POIs are just coordinates attached to images, and only 2 floating point numbers are required to recreate the POI definitions. This enables the storage and sharing of POI definitions without using idiosyncratic data formats with multiple layers of information. For example, our implementation, which can be openly downloaded at https://osf.io/wgma5/, requires only three columns (image name, x-coordinate, y-coordinate) in a CSV file to store each POI information. Researchers could access and examine the POI definitions without having to hassle with various data formats. With the recent emphasis on open science and replicable research across multiple domains [67–71], this simplicity in sharing definitions could be an important advantage of POI-based WSDs over AOI dwell time.

In this article, we defined most of the POIs used for analysis in a data-driven way based on GMM. This data-driven approach could be seen as another advantage of POIs over AOIs since POIs could be guided by the data in this way, while AOIs often have to rely solely on subjective decisions to draw the AOI boundaries. However, this data-driven approach has the drawback of not knowing the POIs prior to the data collection, making it difficult to tailor the design for specific hypothesis testing (e.g., there might be a case where no mean from the GMM component is located near an object of interest). For example, we were not able to find GMM components that matched some of the point of contact AOIs, defined before the experiment, because there was no fixation cluster near the point of contact AOIs. One way of circumventing the issue will be to conduct a small pilot study to ensure that the fixations are clustered near objects of interest, but this could increase the cost of the research. Another way to circumvent this issue is to pick semantically meaningful locations and supplement them to the data-driven POI definitions. However, this approach has the disadvantage of bringing back a lot of subjectivity in POI definitions that the data-driven approach addresses. Additional research will be required to develop a method of defining POIs that could address the issue further such as using computer vision algorithms to define POIs based on semantically relevant objects.

While we showed that our POI-based approach is quite robust, this does not mean that the capabilities of the WSD approach cannot be enhanced. Some hyperparameters could be fine-tuned through additional research. One important hyperparameter in the WSD analysis is the *σ* of the Gaussian kernel. In this study, we set the *σ* of the Gaussian kernels to be 0.75°, but it is uncertain whether this value is the ideal parameter value when examining fixation durations. The approach has to be applied to more datasets to uncover the optimal *σ* value. In addition, it is uncertain that there is a shared *σ* that works well in diverse images with different sizes and objects. More research needs to be conducted to evaluate whether there is a generalizable value that works for most studies, or whether researchers need to calibrate the *σ* for their purposes. Another important hyperparameter is the covariance matrix. In this study, we used an isotropic covariance matrix for the Gaussian kernel so that we were as assumption-free as possible. However, some studies have shown that other forms of the covariance matrix could be more suitable for modeling human fixation patterns [72]. Future research could examine the effect of using other forms of the Gaussian Kernel in applying WSD.

Due to the novelty of using POI in fixation data analysis, there are some limitations to our findings. One important limitation of this work is that we only used one dataset to test the newly proposed method. Though the general concept should apply to other datasets, it is difficult to fully know how generalizable the method will be. We also only tested the Gaussian kernel for weighting in WSD analysis. Although Gaussian kernels are one of the most widely used kernels in various methodology such as smoothing, it will be interesting to exchange the weighting method and see how this affects the results from WSD analysis. Furthermore, the WSD method has only been tested on static images. It is not certain that this method could be readily applied to experiments using non-static stimuli. Moreover, we only compared WSD with AOI dwell time calculated using AOIs defined manually by researchers in this study. We did not investigate how WSD compares to dwell time using automatically generated AOIs. It is possible that WSD works better for certain types of scenes or AOIs than for others.

Another limitation worth mentioning is that while this method retains the interpretability of AOI dwell time and reduces the subjectivity in AOI definitions, it may introduce some issues with regard to interpretability. Since WSDs use sums of transformed fixation durations rather than raw sums of fixation durations, they do not provide intuitive explanations such as “participants fixated on the faces for 300 ms.” If an intuitive explanation is favored for the research purpose, WSDs may be less useful than traditional AOI dwell time analysis. In addition, although the POI method decreases the subjectivity of POI placement to one dimension (location), some subjectivity remains with regard to where the POI should be placed. However, some completely data-driven solutions could be implemented when fixations are clustered an object of interest. Additional research using the proposed method is required to address the above-mentioned limitations and to further validate the POI-based method.

## Conclusion

Weighted Sum Durations analysis based on POIs was proposed as an alternative approach for AOI dwell time analysis. The use of POIs instead of AOIs for the analysis decreases the subjectivity and variability in AOI definitions and accounts for the dichotomous classification problem of AOIs (i.e., whether a fixation falls within an AOI or not if it is right on the border of the AOI, or even very near to it). We checked the robustness of the WSD approach by replicating results from research that used AOI dwell time analysis. The findings of this study provide researchers with a new tool for assessing fixation durations that can be easily replicated and shared across researchers.

## Supporting information

S1 File (PDF)
