# Supplementary material for: Introducing Point-of-Interest as an alternative to Area-of-Interest for fixation duration analysis
Source: PLoS One. 2021 May 10;16(5):e0250170. doi: 10.1371/journal.pone.0250170 (PMC8109773; doi:10.1371/journal.pone.0250170)
Supplement: S1 File — (PDF) [file pone.0250170.s001.pdf]

S1 File: Introducing Point-of-Interest as an alternative to Area-of-Interest for fixation duration analysis

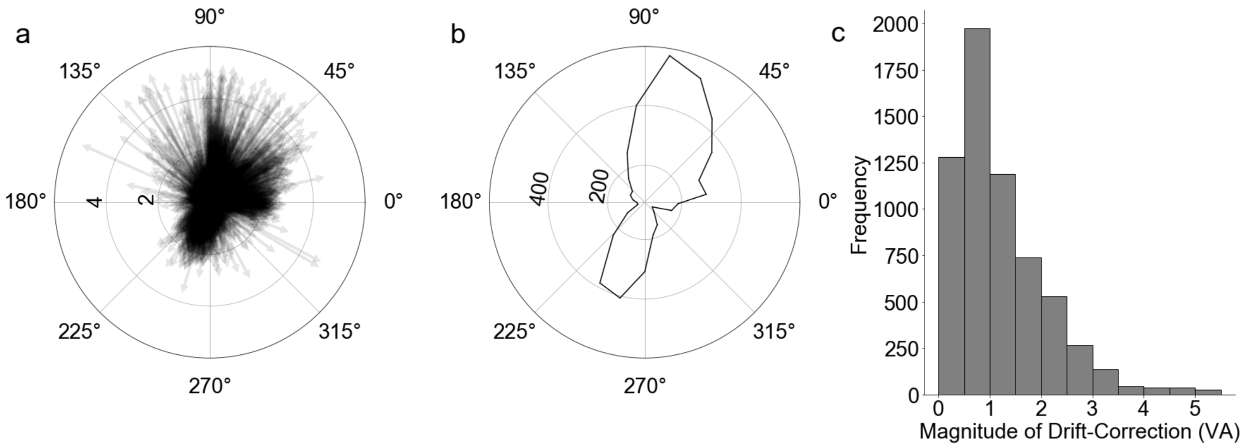

S1 Fig. Direction and magnitude of drift-correction. a) The direction and magnitude of all drift-corrections (outliers removed). The radial distance denotes the magnitude of drift correction, and the polar angle denotes the direction of drift-corrections. b) Radial histogram of directions (angles) of drift-correction. The radial distance denotes the frequency and the polar angle denotes the direction of drift-corrections. c) Histogram of drift-correction magnitude.

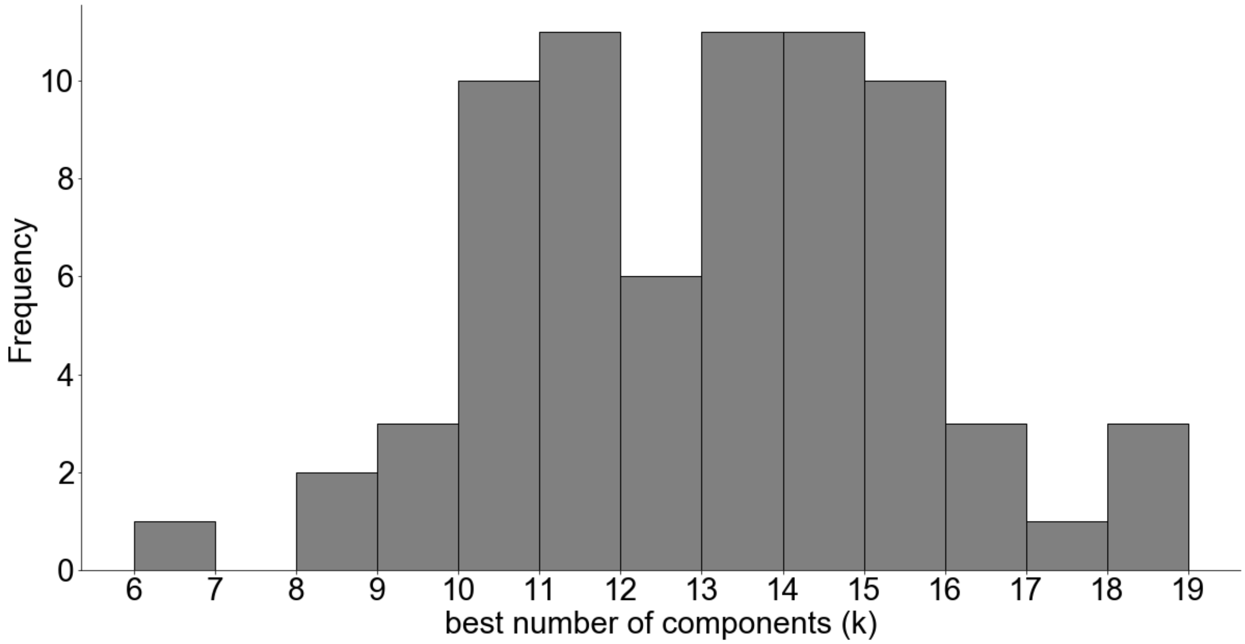

S2 Fig. Distribution of the number of components (*k*) used to fit spherical Gaussian Mixture Models

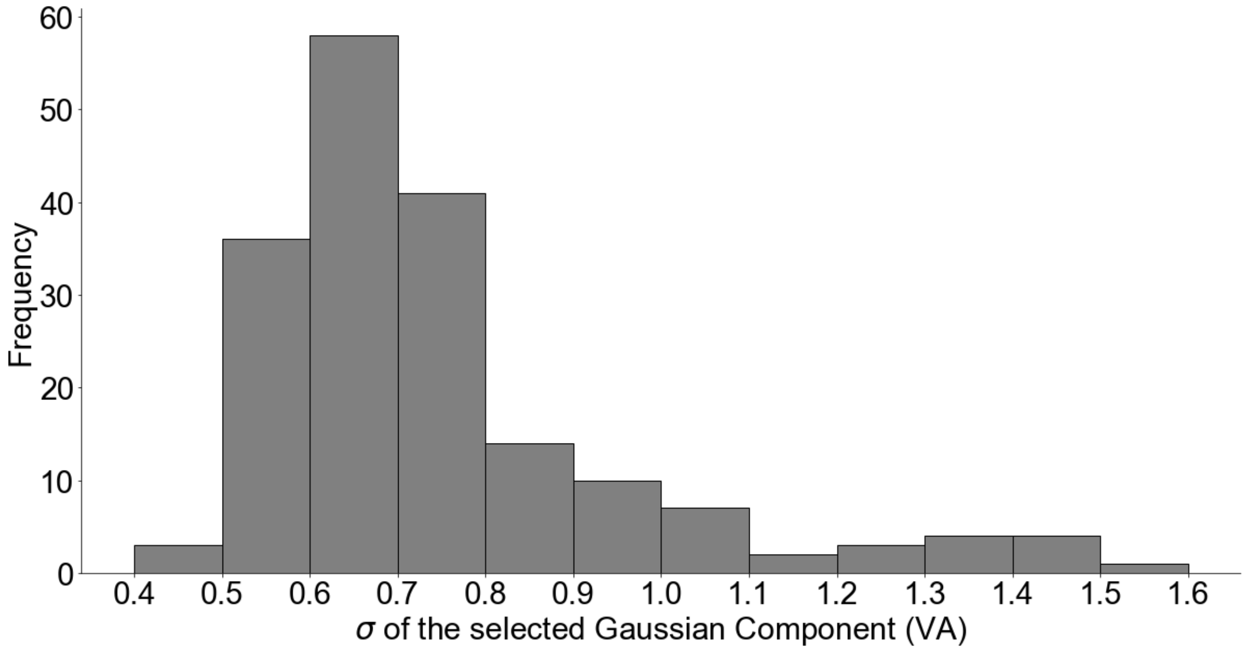

S3 Fig. Distribution of σ of selected components of fitted spherical Gaussian Mixture Models.

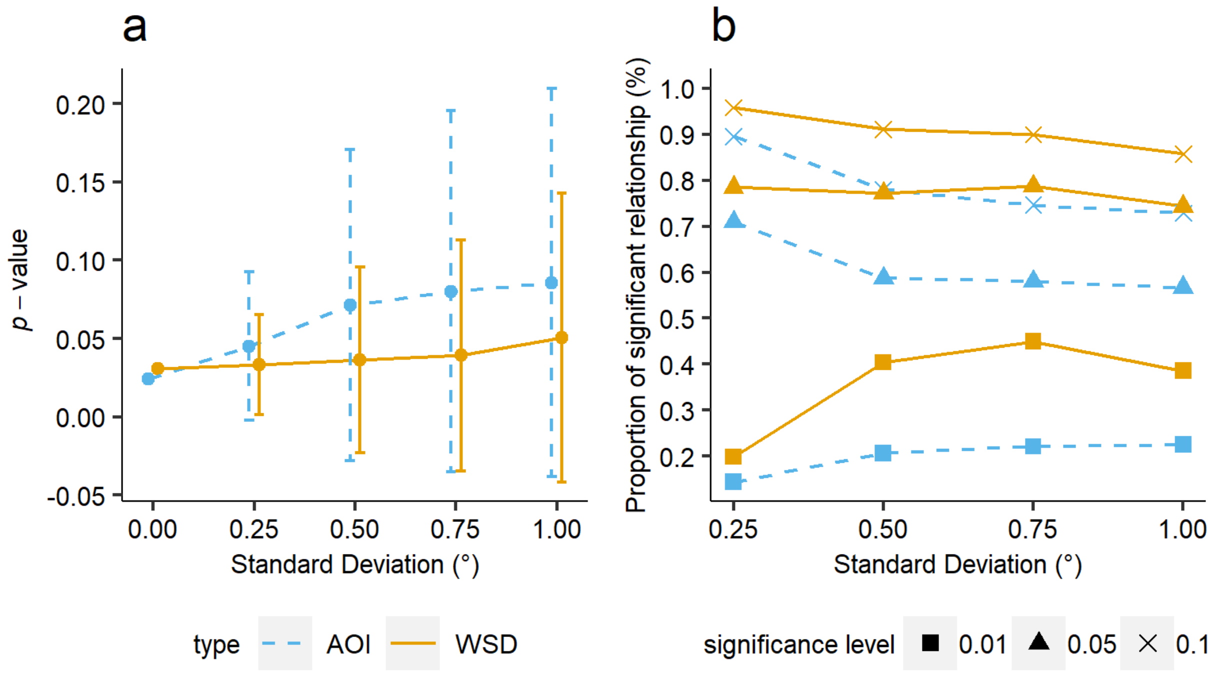

S4 Fig. The effect of noise on LMMs fitted with AOI dwell time and WSD. a) The p-value distribution for LMM fitted with AOI dwell time and WSD across different noise levels. Error bars represent ±1 SD. b) The proportion of LMMs that showed significant relationships between z-scored violence rating and AOI dwell time / WSD on faces across different significance levels and different noise levels.

Model: Dwell Time on Face ~ Interaction Type + AOI Saliency + AOI Size + (1|Participant ID) + (1|Image ID)					
				Marginal R^2 / Conditional R^2: 0.191 / 0.446	
**Predictors**	*b*	Lower CI	Upper CI	*t*	*p*
(Intercept)	2266.534	2074.504	2458.563	23.347	0.000 *
Interaction Type	-360.490	-615.617	-105.379	-2.807	0.006 *
AOI Saliency	146.241	-52.061	344.531	1.465	0.147
AOI Size	285.449	88.125	482.774	2.874	0.005 *
**Random Effects**	**SD**				
Participant ID	408.598				
Image ID	409.049				

Model: Dwell Time on Face ~ Violence Rating + AOI Saliency + AOI Size + (1|Participant ID) + (1|Image ID)					
				Marginal R^2 / Conditional R^2: 0.177 / 0.440	
**Predictors**	*b*	Lower CI	Upper CI	*t*	*p*
(Intercept)	2135.384	2004.478	2266.281	32.192	0.000 *
Violence Rating	-54.662	-102.956	-5.953	-2.253	0.024 *
AOI Saliency	177.351	-23.019	378.454	1.756	0.084
AOI Size	296.380	95.238	497.855	2.926	0.005 *
**Random Effects**	**SD**				
Participant ID	408.527				
Image ID	418.031				

Model: Dwell Time on Point of Contact ~ Interaction Type + AOI Saliency + AOI Size + (1|Participant ID) + (1|Image ID)					
				Marginal R^2 / Conditional R^2 : 0.162 / 0.283	
**Predictors**	*b*	Lower CI	Upper CI	*t*	*p*
(Intercept)	57.155	-73.126	187.402	0.932	0.369
Interaction Type	285.681	110.853	460.538	3.477	0.005 *
AOI Saliency	37.689	-141.467	216.848	0.448	0.662
AOI Size	79.288	-84.317	242.898	1.031	0.323

Random Effects	SD				
Participant ID	62.495				
Image ID	121.465				

Model: WSD on Face ~ Interaction Type + POI Saliency + (1|Participant ID) + (1|Image ID)

Marginal R^2 / Conditional R^2 : 0.030 / 0.333

**Predictors**	*b*	Lower CI	Upper CI	*t*	*p*
(Intercept)	1471.064	1338.960	1603.164	21.995	0.000 *
Interaction Type	-167.365	-327.410	-7.325	-2.077	0.041 *
POI Saliency	94.542	28.693	160.392	2.852	0.006 *
**Random Effects**	**SD**				
Participant ID	346.453				
Image ID	262.224				

Model: WSD on Face ~ Violence Rating + POI Saliency + (1|Participant ID) + (1|Image ID)

Marginal R^2 / Conditional R^2 : 0.022 / 0.327

**Predictors**	*b*	Lower CI	Upper CI	*t*	*p*
(Intercept)	1416.389	1320.820	1511.950	29.237	0.000 *
Violence Rating	-37.803	-72.745	-2.354	-2.157	0.031 *
POI Saliency	104.435	40.571	168.810	3.237	0.002 *
**Random Effects**	**SD**				
Participant ID	346.409				
Image ID	260.958				

Model: WSD on Point of Contact ~ Interaction Type + POI Saliency + (1|Participant ID) + (1|Image ID)

Marginal R^2 / Conditional R^2 : 0.113 / 0.277

**Predictors**	*b*	Lower CI	Upper CI	*t*	*p*
(Intercept)	80.394	-25.027	185.797	1.620	0.131
Interaction Type	177.276	41.676	312.898	2.782	0.017 *
POI Saliency	30.298	-45.428	106.011	0.851	0.411
**Random Effects**	**SD**				
Participant ID	49.730				

Image ID	97.349

S1 Table. Model Statistics for Linear Mixed Models (σ = 0.75°). Marginal R^2, Conditional R^2 is reported for each LMM. *b* estimate, 95% Confidence Intervals, *t*-value, *p*-value are reported for each fixed effect in LMM. The standard deviation of intercepts for each random effect is reported. For interaction type, the effect is for violent images over friendly images.

Model: WSD on Face ~ Interaction Type + POI Saliency + (1|Participant ID) + (1|Image ID)					
			Marginal R^2 / Conditional R^2 : 0.031 / 0.344		
**Predictors**	*b*	Lower CI	Upper CI	*t*	*p*
(Intercept)	1628.908	1487.371	1770.442	22.740	0.000 *
Interaction Type	-176.828	-351.819	-1.843	-2.007	0.048 *
POI Saliency	99.161	27.181	171.141	2.737	0.008 *
**Random Effects**	**SD**				
Participant ID	352.063				
Image ID	287.736				

Model: WSD on Face ~ Violence Rating + POI Saliency + (1|Participant ID) + (1|Image ID)					
			Marginal R^2 / Conditional R^2 : 0.023 / 0.338		
**Predictors**	*b*	Lower CI	Upper CI	*t*	*p*
(Intercept)	1571.737	1471.375	1672.091	30.893	0.000 *
Violence Rating	-39.528	-75.702	-2.915	-2.173	0.030 *
POI Saliency	109.499	39.682	179.814	3.106	0.003 *
**Random Effects**	**SD**				
Participant ID	352.032				
Image ID	286.587				

Model: WSD on Point of Contact ~ Interaction Type + POI Saliency + (1|Participant ID) + (1|Image ID)					
			Marginal R^2 / Conditional R^2 : 0.121 / 0.290		
**Predictors**	*b*	Lower CI	Upper CI	*t*	*p*
(Intercept)	101.985	-14.842	218.791	1.855	0.088
Interaction Type	201.239	50.116	352.392	2.833	0.015 *
POI Saliency	38.531	-46.505	123.550	0.964	0.354
**Random Effects**	**SD**				

Participant ID	54.427
Image ID	108.531

S2 Table. Model Statistics for Linear Mixed Models (σ = 0.85°). Marginal R^2, Conditional R^2 is reported for each LMM. *b* estimate, 95% Confidence Intervals, *t*-value, *p*-value are reported for each fixed effect in LMM. The standard deviation of intercepts for each random effect is reported. For interaction type, the effect is for violent images over friendly images.

Model: WSD on Face ~ Interaction Type + POI Saliency + (1|Participant ID) + (1|Image ID)					
			Marginal R^2 / Conditional R^2 : 0.032 / 0.327		
**Predictors**	*b*	Lower CI	Upper CI	*t*	*p*
(Intercept)	1366.299	1244.460	1488.137	22.137	0.000 *
Interaction Type	-205.501	-348.045	-62.965	-2.864	0.005 *
POI Saliency	85.437	26.821	144.055	2.895	0.005 *
**Random Effects**	**SD**				
Participant ID	345.970				
Image ID	230.243				

Model: WSD on Face ~ Violence Rating + POI Saliency + (1|Participant ID) + (1|Image ID)					
			Marginal R^2 / Conditional R^2 : 0.022 / 0.322		
**Predictors**	*b*	Lower CI	Upper CI	*t*	*p*
(Intercept)	1283.426	1191. 886	1374.962	27.662	0.000 *
Violence Rating	-34.174	-67.723	0.000	-2.040	0.042 *
POI Saliency	100.64	43.065	158.797	3.458	0.001 *
**Random Effects**	**SD**				
Participant ID	345.969				
Image ID	233.691				

Model: WSD on Point of Contact ~ Interaction Type + POI Saliency + (1|Participant ID) + (1|Image ID)					
			Marginal R^2 / Conditional R^2 : 0.111 / 0.260		
**Predictors**	*b*	Lower CI	Upper CI	*t*	*p*
(Intercept)	68.577	-28.117	165.254	1.508	0.157
Interaction Type	167.591	43.204	291.998	2.867	0.014 *
POI Saliency	27.481	-43.177	98.133	0.828	0.424

Random Effects	SD
Participant ID	40.368
Image ID	89.083

S3 Table. Model Statistics for Linear Mixed Models using AOI centers as POIs (σ = 0.75°). Marginal R^2, Conditional R^2 is reported for each LMM. *b* estimate, 95% Confidence Intervals, *t*-value, *p*-value are reported for each fixed effect in LMM. The standard deviation of intercepts for each random effect is reported. For interaction type, the effect is for violent images over friendly images.

Model: WSD on Face ~ Interaction Type + POI Saliency + (1|Participant ID) + (1|Image ID)					
			Marginal R^2 / Conditional R^2 : 0.034 / 0.336		
**Predictors**	*b*	Lower CI	Upper CI	*t*	*p*
(Intercept)	1525.692	1395.229	1656.153	23.093	0.000 *
Interaction Type	-217.336	-373.523	-61.156	-2.764	0.007*
POI Saliency	90.022	25.796	154.251	2.784	0.007*
**Random Effects**	**SD**				
Participant ID	354.043				
Image ID	253.129				
Model: WSD on Face ~ Violence Rating + POI Saliency + (1|Participant ID) + (1|Image ID)					
			Marginal R^2 / Conditional R^2 : 0.023 / 0.330		
**Predictors**	*b*	Lower CI	Upper CI	*t*	*p*
(Intercept)	1440.313	1344.157	1536.464	29.550	0.000 *
Violence Rating	-35.931	-70.942	-0.348	-2.052	0.040 *
POI Saliency	106.458	43.524	169.990	3.348	0.001*
**Random Effects**	**SD**				
Participant ID	354.057				
Image ID	256.644				
Model: WSD on Point of Contact ~ Interaction Type + POI Saliency + (1|Participant ID) + (1|Image ID)					
			Marginal R^2 / Conditional R^2 : 0.118 / 0.275		
**Predictors**	*b*	Lower CI	Upper CI	*t*	*p*
(Intercept)	88.447	-20.030	196.904	1.733	0.108

Interaction Type	190.507	50.230	330.810	2.890	0.014 *
POI Saliency	36.229	-43.825	116.272	0.963	0.355
**Random Effects**	**SD**				
Participant ID	45.541				
Image ID	100.566				

S4 Table. Model Statistics for Linear Mixed Models using AOI centers as POIs (σ = 0.85°). Marginal R^2, Conditional R^2 is reported for each LMM. *b* estimate, 95% Confidence Intervals, *t*-value, *p*-value are reported for each fixed effect in LMM. The standard deviation of intercepts for each random effect is reported. For interaction type, the effect is for violent images over friendly images.
